# The GacS/A-RsmA Signal Transduction Pathway Controls the Synthesis of Alkylresorcinol Lipids that Replace Membrane Phospholipids during Encystment of *Azotobacter vinelandii* SW136

**DOI:** 10.1371/journal.pone.0153266

**Published:** 2016-04-07

**Authors:** Yanet Romero, Josefina Guzmán, Soledad Moreno, Miguel Cocotl-Yañez, Miguel Ángel Vences-Guzmán, Miguel Castañeda, Guadalupe Espín, Daniel Segura

**Affiliations:** 1 Departamento de Microbiología Molecular, Instituto de Biotecnología, Universidad Nacional Autónoma de México, Cuernavaca, Morelos, México; 2 Centro de Investigaciones en Ciencias Microbiológicas, Instituto de Ciencias, Benemérita Universidad Autónoma de Puebla, Puebla, México; University Paris South, FRANCE

## Abstract

*Azotobacter vinelandii* is a soil bacterium that undergoes a differentiation process that forms cysts resistant to desiccation. During encystment, a family of alkylresorcinols lipids (ARs) are synthesized and become part of the membrane and are also components of the outer layer covering the cyst, where they play a structural role. The synthesis of ARs in *A*. *vinelandii* has been shown to occur by the activity of enzymes encoded in the *arsABCD* operon. The expression of this operon is activated by ArpR, a LysR-type transcriptional regulator whose transcription occurs during encystment and is dependent on the alternative sigma factor RpoS. In this study, we show that the two component response regulator GacA, the small RNA RsmZ1 and the translational repressor protein RsmA, implicated in the control of the synthesis of other cysts components (i.e., alginate and poly-ß-hydroxybutyrate), are also controlling alkylresorcinol synthesis. This control affects the expression of *arsABCD* and is exerted through the regulation of *arpR* expression. We show that RsmA negatively regulates *arpR* expression by binding its mRNA, repressing its translation. GacA in turn, positively regulates *arpR* expression through the activation of transcription of RsmZ1, that binds RsmA, counteracting its repressor activity. This regulatory cascade is independent of RpoS. We also show evidence suggesting that GacA exerts an additional regulation on *arsABCD* expression through an ArpR independent route.

## Introduction

*A*. *vinelandii* is a soil bacterium that undergoes a morphological and physiological differentiation process to form cysts that are resistant to desiccation [1]. The cysts have a distinctive morphology, consisting of a contracted oval shaped cell, known as the central body, which contains numerous granules of polyhydroxybutyrate (PHB). The central body is surrounded by a capsule, which is composed of a laminated outer layer called exine, and an inner layer called intine [2]. Cysts are formed by less than 0.01% of late stationary phase cells when grown on carbohydrates; however, by replacing the carbohydrates from exponentially growing cultures with n-butanol or ß-hydroxybutyrate (BHB), the encystment is synchronously induced [2, 3]. During encystment, the synthesis of alkylresorcinols lipids (ARs) and alkylpyrones (APs) is induced and these lipids become part of the membrane and are also structural components of the exine layer of the cyst [3]. The *arsABCD* operon encodes the enzymes that synthesize these compounds [4]. ArsB and ArsC proteins are type III polyketide synthases, which synthesize ARs and APs respectively, whereas ArsA is a fatty acid synthase responsible for the synthesis of the C23-C25 fatty acids that, together with malonyl-CoA, serve as substrates for ArsB and ArsC [5]. ArsD is a 4´-phosphopantetheinyl transferase that would catalyze the postranslational modification of ArsA [4, 5].

Expression of the *arsABCD* operon is very low in exponentially growing vegetative cells and increases 14 fold during stationary phase, when a low percentage of encystment occurs; however, under encystment induction conditions its expression is induced 200-fold [2]. For this induction the ArpR protein is needed. ArpR is a LysR-type transcriptional regulator that activates transcription of the *arsABCD* operon during encystment. The activation of transcription of *arsABCD* by ArpR is dependent on a metabolic signal, acetoacetyl-CoA, which acts as a coinducer [6]. The alternative sigma factor RpoS was also shown to be involved in the control of ARs synthesis, because an *rpoS* mutant is unable to synthesize these lipids [7]. This regulation of ARs synthesis by RpoS was later demonstrated to occur through the control of transcription of the *arpR* gene [6]. Another regulatory system involved in the control of ARs synthesis is the nitrogen-related phosphotransferase system (PTS^Ntr^), a global regulatory system comprised by three proteins, EI^Ntr^, NPr and EIIA^Ntr^. These proteins participate in a phosphoryl-group transfer from phosphoenolpyruvate to EIIA^Ntr^ via the phosphotransferases EI^Ntr^ and NPr. The non-phosphorylated EIIA^Ntr^ protein was shown to negatively affect the activation of transcription of the regulatory *arpR* gene by RpoS [8].

In *A*. *vinelandii* the two component regulatory system integrated by the GacS sensor kinase and the response regulator GacA, regulates the synthesis of two cyst components, alginate and polyhydroxybutyrate. The control of the synthesis of these polymers is achieved by regulating in turn a post-trancriptional regulatory system, consisting of the protein called RsmA and the small RNAs named RsmZ1-7 and RsmY. The RsmA protein binds to the *m*RNAs of the *algD* and *phbR* genes, acting as a translational repressor. The RsmZ-Y RNAs bind RsmA, counteracting its repressor activity. The role of GacA in this regulatory system is to activate the transcription of the genes encoding the RsmZ and RsmY RNAs [9, 10].

In this study we show that the synthesis of ARs is also regulated by the Gac system. A mutation in *gacA* impairs the synthesis of ARs. We demonstrate that this phenotype is due to an effect on the expression of *arpR*, negatively affecting in turn the transcription of the *arsABCD* biosynthetic operon. We demonstrate that the Gac system acts trough its regulation of the Rsm system, which postranscriptionally represses *arpR* by binding of RsmA to its mRNA. Inactivation of *rsmA* in the *gacA* mutant restored the expression of *arpR*, as expected according to our regulatory model, but neither transcription of *arsA* nor alkylresorcinol synthesis were reestablished. The constitutive expression of the ArpR regulator, reestablished ARs production in the *rsmZ1* mutant, but not in the *gacA*, nor in the *gacA-rsmA* double mutant, constituting evidence for the existence of an additional regulation of *arsA* by GacA which is independent of the Rsm system and probably also of ArpR in the presence of its known conducer acetoacetyl-CoA.

## Materials and Methods

### Bacterial strains, media and growth conditions

Bacterial strains and plasmids used in this work are listed in S1 Table. Liquid cultures were carried out in 250 ml flasks containing 50 ml of medium, in a rotatory shaker at 250 rpm and 30°C. Vegetative cells were obtained by growing *A*. *vinelandii* on Burk´s medium [11], supplemented with 2% sucrose (BS). The inocula were grown on BS, washed twice with Burk´s medium without carbon source (Burk´s buffer) and transferred to the indicated medium. *E*. *coli* strains were grown at 37°C on Luria Bertani medium (LB). Antibiotic concentrations routinely used were as follows: nalidixic acid, 20 μg/ml; rifampicin, 10 μg/ml; kanamycin, 2 μg/ml; spectinomycin, 25 μg/ml, gentamicin, 0.25 μg/ml.

### Encystment induction

Cyst formation was induced by transferring vegetative cells grown for 24 h on BS, to flasks or plates (as indicated) containing Burk´s medium with 0.2% butanol instead of sucrose as carbon source (encystment induction medium, BOH) [1]. After 5 days of incubation at 30°C, the cysts were collected and were suspended in Burk’s medium without carbon source (Burk’s buffer) for ARs staining and quantification.

### Visualization of ARs production by staining on Petri dishes

For visualization of the ARs production phenotype, these phenolic lipids were stained with Fast Blue B. The *A*. *vinelandii* strains were grown for 5 days on encystment induction medium (BOH) to induce encystment. The bacterial colonies on the Petri dishes were then sprayed with a solution of Fast Blue B 0.5% in 5% acetic acid. ARs producing colonies turned dark red after a few minutes of reaction with the staining solution [2].

### Determination of alkylresorcinols

For quantification, ARs lipids were extracted with acetone for 20 minutes at room temperature in closed tubes. After centrifugation, the acetone extract was removed, and a second extraction was done with acetone for 12 h at room temperature. The resulting extracts were mixed and used for the spectrophotometric determination of alkylresorcinols, with the use of Fast Blue B as previously described [12]. Orcinol (3,5-dihydroxytoluene; Merck) was used as standard. The protein content of the cells used for ARs determination was quantified by the method of Lowry *et al*. [13].

### DNA manipulations

For the isolation of total genomic DNA, restriction endonuclease digestion, agarose gel electrophoresis, purification of DNA from agarose, DNA ligations and transformation of *E*. *coli* DH5, standard procedures were carried out, as described by Sambrook *et al*. [14]. DNA sequences were determined by the dideoxy chain termination method [15]. For Southern blot analysis, DNA samples were digested with the indicated restriction endonucleases, DNA fragments were separated in 1% agarose gel and blotted as described by Sambrook *et al*. [14]. The radioactive probes were prepared by random priming using the Rediprime DNA labelling system (GE Healthcare).

### Construction of *A*. *vinelandii* mutants *gacA*^-^, *rsmA*^-^, *rsmZ1*^-^ and *gacA*^-^ *rsmA*^-^

To construct a *gacA*^-^ mutant derivative of *A*. *vinelandii* SW136, competent cells of this strain were transformed with total DNA from the *A*. *vinelandii* mutant JM3, which is a derivative of strain ATCC 9046 that contains a gentamicin resistance cassette inserted within *gacA* [16]. A transformant of strain SW136, resistant to gentamicin, was isolated and named SW5 (S1 Table). The replacement of the *gacA* gene was confirmed by a PCR analysis carried out with primers GacA-U (5´ cga ttc tcc tga cag ttc 3´) and GacA-L (5´cgg aaa tag ctg gac aag 3´) that flank the insertion site.

The *rsmA*^-^ mutant was generated using total DNA from strain AHrsmA, a UW136 mutant derivative which contains a spectinomycin resistance cassette inserted into the *rsmA* gene [9]. This DNA was used to transform strain SW136 and a spectinomycin resistant transformant was isolated and confirmed to contain the inactivated *rsmA*::Sp^r^ allele by PCR analysis using primers RSMA2D (5´ ggc cgg gca ccc tca tta cca 3´) and RSMA2R (5´ aac tcg ccg ctt cgc cca tct tat c 3´) [9]. This isolate was named SW11 (S1 Table).

The *rsmZ1*^-^ mutant was constructed by transforming SW136 competent cells with total DNA purified from the *rsmZ1*::Km^r^ mutant strain AEIV*rsmZ1* [10]. A kanamycin resistant transformant was confirmed to contain the *rsmZ1*::Km^r^ gene inactivation also by PCR analysis using primers RSMZ1-2D (5´ cga agg cgg aca ggc tca g 3´) and RSMZ1-2R (5´ atc ggg cgg cgc ggc ttt ca 3´)[10]. This isolate was named SW13 (S1 Table).

To construct a double mutant containing both the *gacA*::Gm^r^ and the *rsmA*::Sp^r^ gene inactivations, competent cells of mutant SW5 were transformed with total DNA from strain AHrsmA [9]. A transformant resistant to spectinomycin was named SW15 (S1 Table). This isolate was confirmed to contain the *rsmA*::Sp^r^ mutation by PCR analysis using primers RSMA2D and RSMA2R [9].

### Construction of strains carrying chromosomal *arsA*::*gusA* and *arpR*::*gusA* transcriptional fusions

To introduce *arsA*::*gusA* or *arpR*::*gusA* transcriptional fusions into the chromosomes of the *gacA*^-^, *rsmA*^-^ and *rsmZ1*^-^ mutants, we used plasmids pYRR50 and pYRR52 [6] respectively. These suicide plasmids contain the intergenic regulatory region and part of the coding regions of *arsA* and *arpR* respectively, transcriptionally fused to the ß-glucuronidase gene *gusA*. They also contain a spectinomycin resistance cassette [6]. The *gacA*^-^, *rsmA*^-^ and *rsmZ1*^-^ mutant derivatives containing the *arsA*::*gusA*-Sp^r^ transcriptional fusion, were constructed transforming the corresponding *A*. *vinelandii* mutants SW5, SW11 and SW13 with plasmid pYRR50. Spectinomycin resistant transformants were selected, resulting strains YRR36, YRR38, and YRR40, respectively (S1 Table). These strains were confirmed to contain the *arsA*::*gusA*-Sp^r^ gene fusion by PCR analysis using primers UIARS (5´ atg ctt cta gag tct gtg ctg atc cat 3´) and SPlow (5´gct tta tgc ttg taa tcc 3´). For the construction of the *gacA*^-^, *rsmA*^-^ *and rsmZ1*^-^ mutant strains containing the *arpR*::*gusA*-Sp^r^ transcriptional fusion, mutants SW5, SW11 and SW13 were transformed with plasmid pYRR52. Spectinomycin-resistant transformants containing the transcriptional fusion were isolated, producing strains YRR54, YRR56, and YRR58 respectively (S1 Table). These strains were confirmed to contain the *arpR*::*gusA*-Sp^r^ transcriptional fusion cointegrated into the chromosome by PCR analysis using primers PwarsR (5´ atc tgg atc cat ggg gaa ggc cta tcc 3´) and SPlow (5´gct tta tgc ttg taa tcc 3´).

### Construction of strains carrying chromosomal *arsA*::*gusA* and *arpR*::*gusA* translational fusions

To construct translational gene fusions of both *arsA* and *arpR* with the ß-glucuronidase reporter gene *gusA*, a DNA fragment containing *gusA* without ribosome binding site and a spectinomycin resistance cassette, was amplified by PCR using plasmid pMP01 as template [17] and primers UGA2 (5´ ctt atg tta cgt cct gta gaa acc cca ac 3´) and SPlow (5´ gct tta tgc ttg taa tcc 3´). This fragment was cloned into pMOSBlue, and the resulting plasmid was named pMOSgA. In the case of the translational fusion of *arsA* with *gusA*, plasmid pYRR40 [6], containing the intergenic regulatory region and part of the coding region of *arsA*, was digested with *Bgl*II and *Apa*I. Plasmid pMOSgA was digested with the same enzymes and the resulting fragment containing *gusA* was ligated with the digested pYRR40. The resulting plasmid containing the *arsA*-*gusA*-Sp^r^ gene fusion was named pYRR51. The SW136 wild type and the *gacA*, *rsmZ1* and *rsmA* mutant strains were transformed with this suicide plasmid and spectinomycin resistant transformants were selected and named YRR31, YRR33, YRR41 and YRR39 respectively (S1 Table). The presence of the translational gene fusion of *arsA-gusA*, generated by cointegration of the plasmid, was confirmed by PCR analysis of all these transformants using primers UIARS (5´ atg ctt cta gag tct gtg ctg atc cat 3´) and SPlow (5´gct tta tgc ttg taa tcc 3´).

The translational gene fusion of *arpR* with *gusA* was constructed using plasmid pYRR41 [6], that carries the intergenic regulatory region and part of the coding sequence of *arpR*. It was digested with *Bam*HI and *Hind*III and ligated with the DNA fragment containing *gusA* resulting from the digestion of pMOSgA with the same enzymes. The plasmid produced, which contains the translational *arpR*-*gusA*-Sp^r^ gene fusion, was named pYRR53. The SW136 wild type and the *gacA*, *rsmZ1* and *rsmA* mutant strains were transformed with this plasmid and the corresponding spectinomycin resistant isolates were shown to contain the chromosomal *arpR-gusA* translational fusion by PCR analysis using primers PwarsR (5´ atc tgg atc cat ggg gaa ggc cta tcc 3´) and SPlow (5´gct tta tgc ttg taa tcc 3´). These strains were named YRR51, YRR53, YRR59, YRR57 respectively (S1 Table).

### ß-glucuronidase activity determinations

ß-glucuronidase activity was measured as reported by Miller; 1 U corresponds to 1 nmol of p-nitrophenyl-β-D-glucuronide hydrolyzed per min per mg of protein. Protein was determined by the method of Lowry *et al*. [13]. One unit of ß-glucuronidase corresponds to 1 nmol of substrate (X-Gluc) hydrolyzed min^-1^ mg Protein^-1^.

### Real-Time PCR

Expression of *arsA* and *arpR* was measured by qRT-PCR, as previously reported [6]. Total RNA extraction was performed as reported by Barry *et al*. [18]. To eliminate genomic DNA, RNA was treated with DNase (DNA-free^™^, Ambion) and its concentration measured by 260/280 nm ratio absorbance. cDNA was synthesized using 500 ng of DNase-treated total RNA, the Revert Aid^™^ H First Strand cDNA Synthesis kit (Thermo Scientific), and 5 pmol of the specific reverse primers. The cDNA obtained was used as template for Real-Time PCR assays. The primers used [6] were as follows: arsA-RT-F (5´ cac cct cgt caa tct gct c 3´) and arsA-RT-R (5´ gat cct ggt cga aga cct tg 3´) for *arsA*; arpR-RT-F (5´ ctt ccc ctg ctg gca ctc 3´) and arpR-RT-R (5´ cgt tcc tgg agt tct tcg ag 3´) for *arpR*; and fw-gyrA (5´ cca gca agg gca agg tct a 3´) and rev-gyrA (5´ tcg tcc agc ggc aac agg t 3´) for *gyrA*, which was used as internal control in the same samples to normalize the results obtained. The size of all amplimers was 100–101 bp. All real-time PCR reactions were performed in triplicate for each gene of each strain. The quantification technique used to analyze the data was the 2 ^hi,ΔCT^ method reported by Livak and Shmittgen [19]. Reproducibility of the whole procedure was determined by performing cDNA synthesis and Real—Time PCR experiments from two separate RNAs extracted for each strain. Similar results were obtained for the transcription of all measured genes in the repetitions and with the internal control (*gyrA*) used for the normalization.

### Purification of His6-RsmA protein

The His6-RsmA protein was expressed from a plasmid pYG1 in TOP10 *E*. *coli* cells, and it was purified as previously described by Manzo *et al*. [10] and was concentrated using a Microcon YM-3 centrifugal filter unit (Amicon) at 4°C. Protein concentration was estimated by the Lowry method with BSA as standard [13].

### *In vitro* transcription of the *arpR* leader

A 290 bp fragment corresponding to the leader region of *arpR*, including the SD sequence, was amplified by PCR using primers ARRA-Up (5´ agt cgg atc cgg gta tcc ata tgg gga ag 3´) and ARRA-Lw (5´ gat caa gct tct ccg cct tgt acc aag aga 3´). The resulting PCR product was digested with *Hind*III and *Eco*RI and ligated into plasmid pTZ19R (ThermoScientific), previously digested with the same enzymes, to produce plasmid pARRA. Plasmids pARRA and pLM1 [9] were used as templates to amplify the DNA fragments containing *arpR* and *sodB* regulatory regions under the control of the T7 promoter. These PCR products were used to generate the *arpR* and *sodB* radioactively labeled transcripts *in vitro* using the TranscriptAid T7 Transcription Kit (ThermoScientific), following the manufacturer’s instructions, in the presence of [α-32P]CTP (PerkinElmer). Unlabeled RNAs were synthesized following the same protocol but with unlabelled CTP. RNA concentrations were estimated by UV absorption at 260 nm.

### Gel mobility shift assays

The gel mobility shift assays were carried out as described by Valverde *et al*. [20] and Manzo *et al*. [10] with some modifications. Binding reactions contained 25 mM Tris-HCl at pH 7.5, 20 mM KCl, 0.5 mM EDTA, 0.5 mM DTT, 5% (w/v) glycerol, 32.5 ng of yeast tRNA, 4 U of RNAse inhibitor (Roche), 10 nM of the RNA generated by *in vitro* transcription, labelled with [α-32P]CTP, and purified His6-RsmA at various concentrations. Reaction mixtures were incubated for 45 min at 30°C to allow complex formation. Samples were then fractionated on native 6% polyacrilamide gels and radioactive bands were detected by autoradiography.

### Statistical analysis

Data were analyzed by unpaired Student’s *t* tests by using GraphPad Prism version 6.0 (GraphPad Software Inc).

## Results

### Alkylresorcinol biosynthesis is under the control of GacA and the Rsm system

In *A*. *vinelandii* the GacS/A system controls expression of genes involved in the synthesis of alginate and polyhydroxybutyrate, two polymers present in mature cysts. This regulation is exerted through the activation of expression of the RsmZ/Y small RNAs that titrate the RsmA protein, that acts as a translational repressor of genes involved in the synthesis of these polymers [9, 10]. In order to investigate whether the Gac-Rsm system controls synthesis of alkylresorcinol lipids during encystment, we determined the production of ARs in strains carrying mutations in *gacA*, *rsmZ1* and *rsmA* genes.

**Fig 1:**
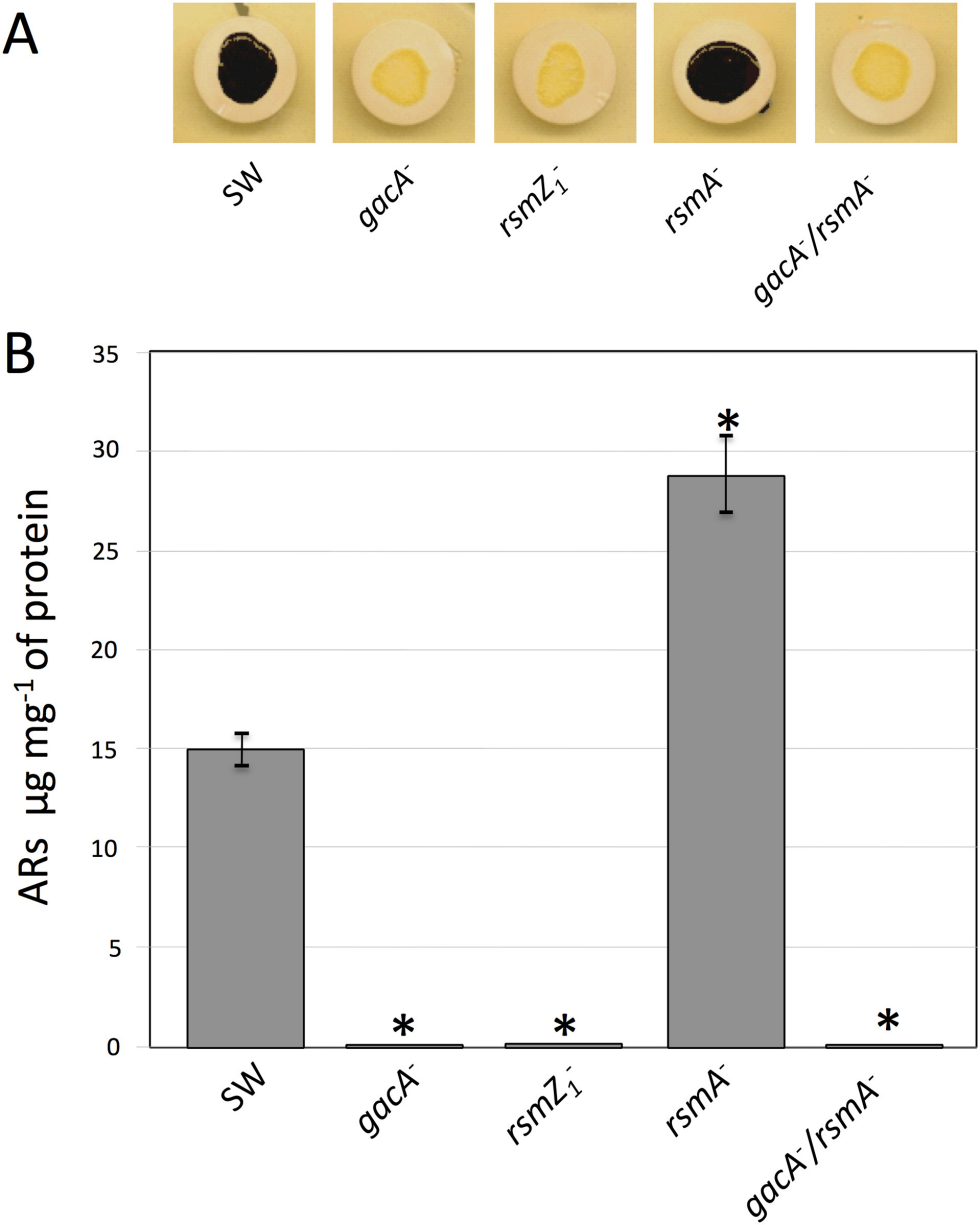
Production of ARs by different *A*. *vinelandii* mutants. (**A**) Staining with Fast Blue B of the ARs produced by colonies of *A*. *vinelandii* strain SW136 (Wild type), and its mutant derivatives inactivated in the genes *gacA*, *rsmZ1*, *rsmA* (strains SW5, SW13 and SW11) and the double mutant *gacA*-*rsmA* (SW15). The colonies were grown on Burk-Butanol (encystment induction medium) for 5 days prior to staining. (**B**). Quantification of the ARs produced by the mutants under the same conditions. The data represent the mean of triplicates and the error bars represent the standard deviations. Asterisks indicate statistical significance (unpaired Student's *t*-test) in the comparison of each mutant versus the wild type strain, *P < 0.05.

The production of ARs was analyzed under encysting inducing conditions. The presence of these lipids was visualized by staining the colonies of *A*. *vinelandii* with Fast Blue B, which turns ARs dark red [2]. As can be seen in Fig 1A, the *gacA* mutant remained unstained. The quantification of the ARs produced confirmed that this mutation abrogated ARs production (Fig 1B). A similar result was obtained for the *rsmZ1* mutant, whereas the *rsmA* mutant showed a production of ARs increased by 80%, as compared to the wild type strain SW136. These results suggested that GacA is a regulator of the biosynthesis of these lipids in *A*. *vinelandii* and that this regulation could be exerted through the control of the Rsm system, in a similar way to that demonstrated for alginate and PHB synthesis.

Because the activation of expression of the RsmZ-Y RNAs by GacA was demonstrated for cells under vegetative growth conditions [9], we first confirmed the regulation of RsmZ1 by GacA under encysting inducing conditions by analyzing the effect of the *gacA* mutation on expression of a chromosomal *rsmZ1-gusA* gene fusion. The level of expression of *rsmZ1* in the *gacA* mutant at different times after induction of encystment on n-butanol (12, 24, 36 and 48 h), diminished more than 90% with respect to that of the strain with the wild type *gacA* gene, in a similar way to what is observed in vegetative cells of the *gacA* mutant, confirming the regulation of this small RNA by GacA in the cysts (S1 Fig).

In a different *A*. *vinelandii* strain (ATCC 9046), the GacS/A system also controls the expression of the alternative sigma factor RpoS [16], which has been shown to regulate the expression of ArpR, the activator of the *ars* biosynthetic genes in *A*. *vinelandii* SW136 [6]; therefore, we also investigated the role of GacA on the expression of RpoS in this strain to establish if the effect of the *gacA* mutation on ARs synthesis could be due to a diminished *rpoS* expression. The quantification of the mRNAs of *rpoS* by qRT-PCR in *A*. *vinelandii* SW136 wild type and in its *gacA* mutant showed no differences in the expression levels (data not shown), demonstrating that the GacA regulator does not participate in the control of *rpoS* expression in this strain. As a control, the *A*. *vinelandii* wild type strain ATCC 9046 and its *gacA* mutant named JM3 [16], were simultaneously analyzed. The mRNAs of *rpoS* in this mutant were diminished 75%, confirming the regulatory effect of GacA on *rpoS* expression previously reported for this strain [16]. These result show that the control of the GacS/A system on ARs synthesis is probably exerted through the control of the Rsm system and not through RpoS.

### Inactivations of components of the GacA-Rsm system affect the level of the *arsA* and *arpR* transcripts

**Fig 2:**
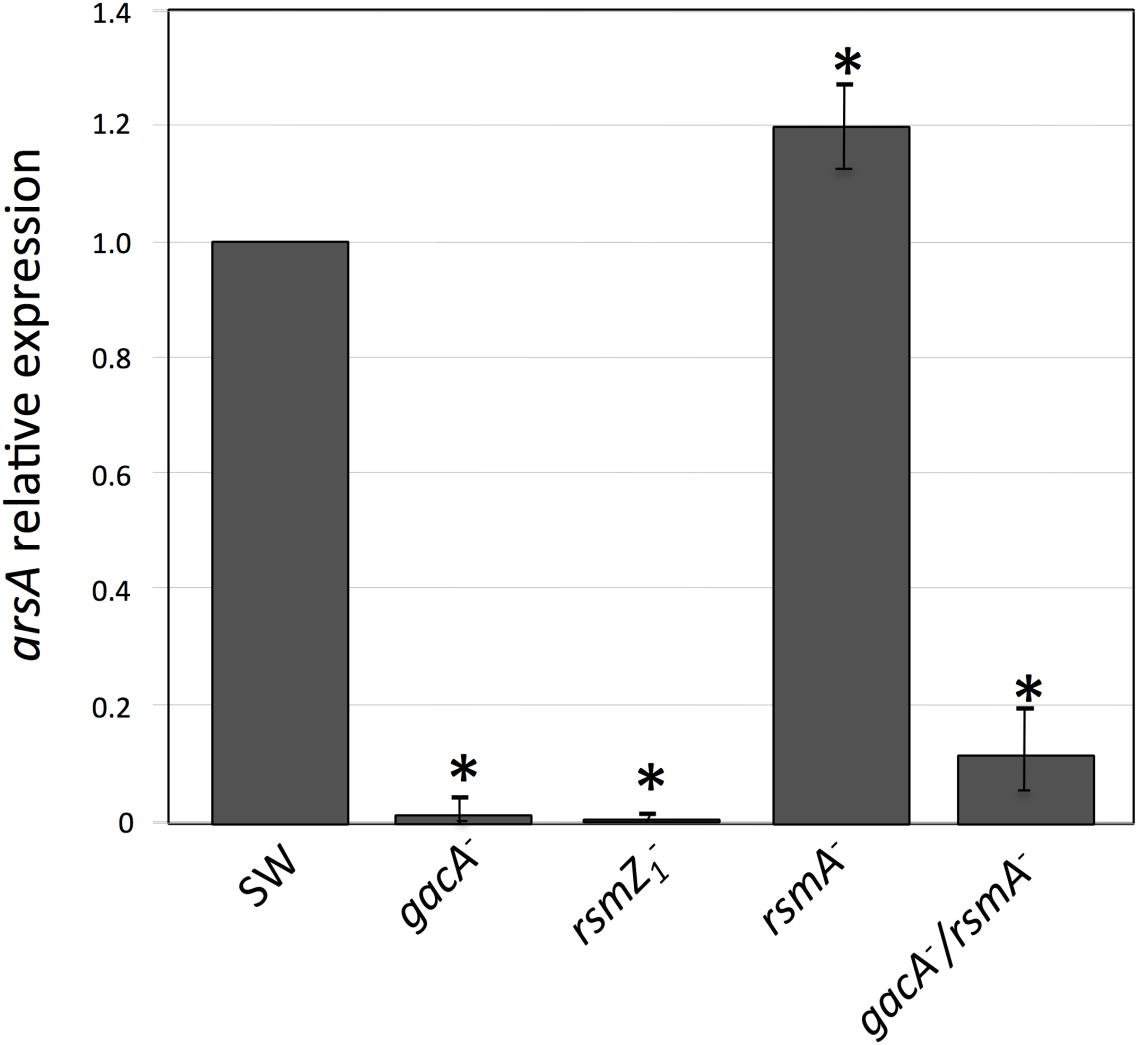
Effect of different gene inactivations on *arsA* gene expression measured by RT-qPCR. The level of the *arsA* transcripts was measured under encystment inducing conditions and it was normalized according to the level of the *gyrA* mRNA. The data are presented as fold change of *arsA* mRNA levels in mutants with *gacA*, *rsmZ1*, *rsmA*, and *gacA*-*rsmA* gene inactivations (strains SW5, SW13, SW11 and SW15), relative to those of the wild type strain (SW136). Determinations were made from bacterial cultures grown for 36 h in liquid BBOH medium at 30°C. The data represent the mean of triplicates and the error bars represent standard deviations. Asterisks indicate statistical significance (unpaired Student's *t*-test) in the comparison of each mutant versus the wild type strain, *P < 0.05.

To further investigate the role of GacA, RsmA and RsmZ1 on the regulation of ARs synthesis, we determined the effect the *gacA*, *rsmZ1* and *rsmA* gene inactivations on the expression of the ARs biosynthetic gene *arsA* by RT-qPCR. In agreement with the results of ARs production, a strong reduction in the level of transcripts was observed in the *gacA* and *rsmZ1* mutants, whereas in the *rsmA* mutant the level of mRNAs was 20 percent higher than in the wild type strain (Fig 2).

**Fig 3:**
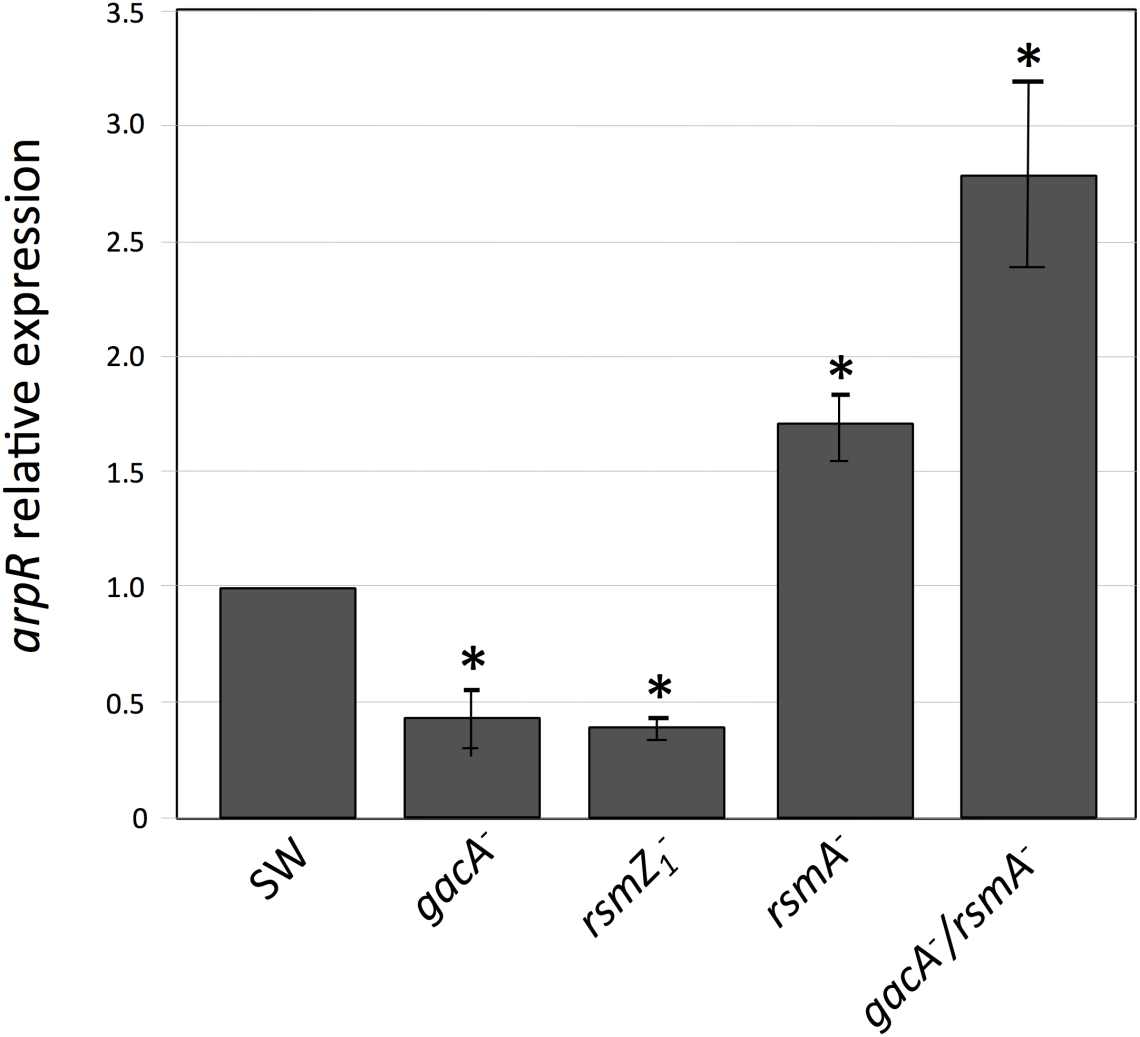
Effect of different gene inactivations on *arpR* gene expression measured by RT-qPCR. The level of the *arpR* transcripts was measured under encystment inducing conditions and it was normalized according to the level of the *gyrA* mRNA. The data are presented as fold change of *arpR* mRNA levels in mutants with *gacA*, *rsmZ1*, *rsmA*, and *gacA*-*rsmA* gene inactivations (strains SW5, SW13, SW11 and SW15), relative to those of the wild type strain (SW136). Determinations were made from bacterial cultures grown for 36 h in liquid BBOH medium at 30°C. The data represent the mean of triplicates and the error bars represent standard deviations. Asterisks indicate statistical significance (unpaired Student's *t*-test) in the comparison of each mutant versus the wild type strain, *P < 0.05.

To determine whether the effects of the *gacA*, *rsmZ1* and *rsmA* mutations on ARs synthesis and on *arsA* expression are exerted through the regulation of ArpR, the transcriptional activator of the ARs biosynthetic operon *arsABCD* [6], the transcripts of the *arpR* gene were also analyzed in the mutants (Fig 3). The mRNAs of *arpR* were 55 and 60 percent lower in the *gacA* and *rsmZ1* mutants respectively, and increased 65 percent in the *rsmA* mutant. These results support a model were the control of the GacS/A system on ARs synthesis is probably exerted through the control of the Rsm system, which in turn regulates the expression of the gene of the activator of the biosynthetic operon ArpR.

### Post-transcriptional regulation of *arpR*

Because the RsmA protein has been shown to regulate translation of the mRNAs of its target genes [21, 22, 23], we analyzed the effect of *gacA*, *rsmA* and *rsmZ1* gene inactivations on expression of *arsA* and *arpR* using transcriptional and translational *arsA-gusA* and *arpR-gusA* gene fusions.

**Fig 4:**
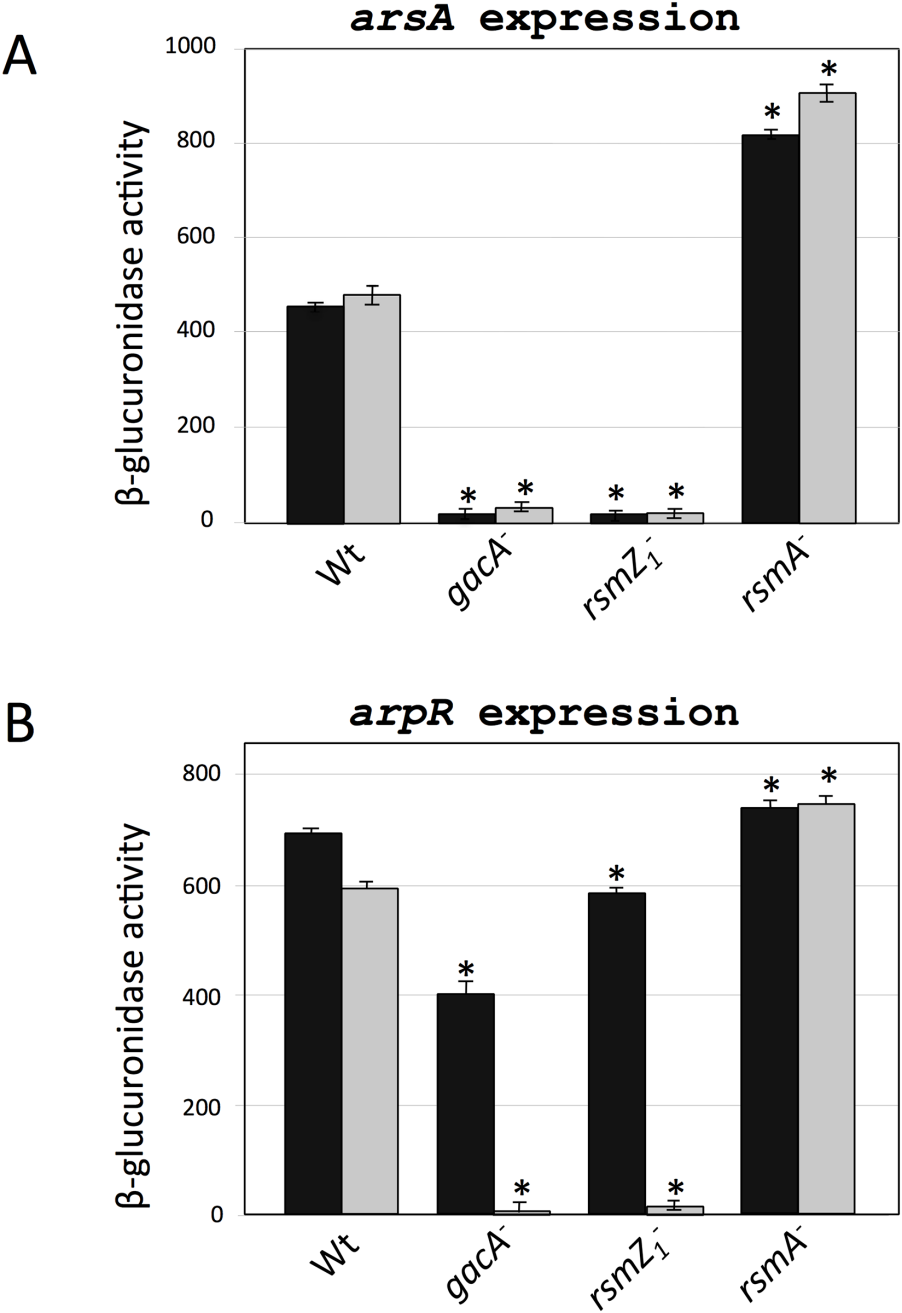
Effect of *gacA*, *rsmZ1*, *rsmA* gene inactivations on the expression of the ARs biosynthetic gene *arsA* and its regulatory gene *arpR* measured using chromosomal transcriptional and translational gene fusions. Expression levels of *arsA* (**A**) and *arpR* (**B**) were quantified as ß-glucuronidase activity of strains carrying *arsA*::*gusA* and *arpR*::*gusA* transcriptional (black bars) and translational (grey bars) gene fusions. The strains used for *arsA* gene expression determinations were YRR30, YRR36, YRR40 and YRR38 (Wild type and its *gacA*, *rsmZ1* and *rsmA* mutant derivatives containing an *arsA*::*gusA* **transcriptional** gene fusion respectively); and YRR31, YRR33, YRR41, YRR39 (Wild type and its *gacA*, *rsmZ1* and *rsmA* mutant derivatives containing an *arsA*::*gusA* **translational** gene fusion). For *arpR*, the strains were YRR50, YRR54, YRR58, and YRR56 (Wild type and its *gacA*, *rsmZ1* and *rsmA* mutant derivatives containing an *arpR*::*gusA* **transcriptional** gene fusion respectively); YRR51, YRR53, YRR59 and YRR57 (Wild type and its *gacA*, *rsmZ1* and *rsmA* mutant derivatives containing the *arpR*::*gusA* **translational** gene fusion). One unit of ß-glucuronidase corresponds to 1 nmol of substrate (X-Gluc) hydrolyzed min^-1^ mg Protein^-1^. Determinations in both panels were made from bacterial cultures grown for 36 h in liquid BBOH medium at 30°C. Error bars represent standard deviations. Asterisks indicate statistical significance (unpaired Student's *t*-test) in the comparison of each mutant versus the corresponding wild type strain with the same gene fusion, *P < 0.05.

The ß-glucuronidase activity of strains derivatives of SW136, *gacA*, *rsmZ1* and *rsmA* strains carrying transcriptional and translational fusions of *arsA*, were very similar between them (Fig 4A) and showed the same effects observed for the *arsA* expression determinations obtained by RT-qPCR (Fig 2). The *rsmZ1* and *gacA* mutants showed a considerably diminished ß-glucuronidase activity, whereas the *rsmA* mutant showed an activity level increased by 75%. This results suggest that the effect on *arsA* is at the transcriptional level.

With respect to the effects of the *gacA* and *rsmZ1* inactivations on the ß-glucuronidase activities of the strains carrying the *arpR-gusA* fusions (Fig 4B), these were considerably stronger for the translational than for the transcriptional fusion. Translation of *arpR* was considerably diminished in both mutants. The *rsmA* mutation had a slight positive effect on the expression of both fusions.

The results are compatible with a regulatory model where the RsmA protein would be a negative translational regulator of the *arpR* mRNA, and also would have a negative effect on the stability of the *arpR* transcripts, as has been demonstrated for other target mRNAs in *A*. *vinelandii* [9]. This would explain the observed reduction of *arpR* transcripts in the *gacA* and *rsmZ1* mutants, quantitated by RT-qPCR, to about 40% and 35% of the wild type and the 60% higher level in the *rsmA* mutant.

### RsmA binds to the 5´ leader of the *arpR* transcript

**Fig 5:**
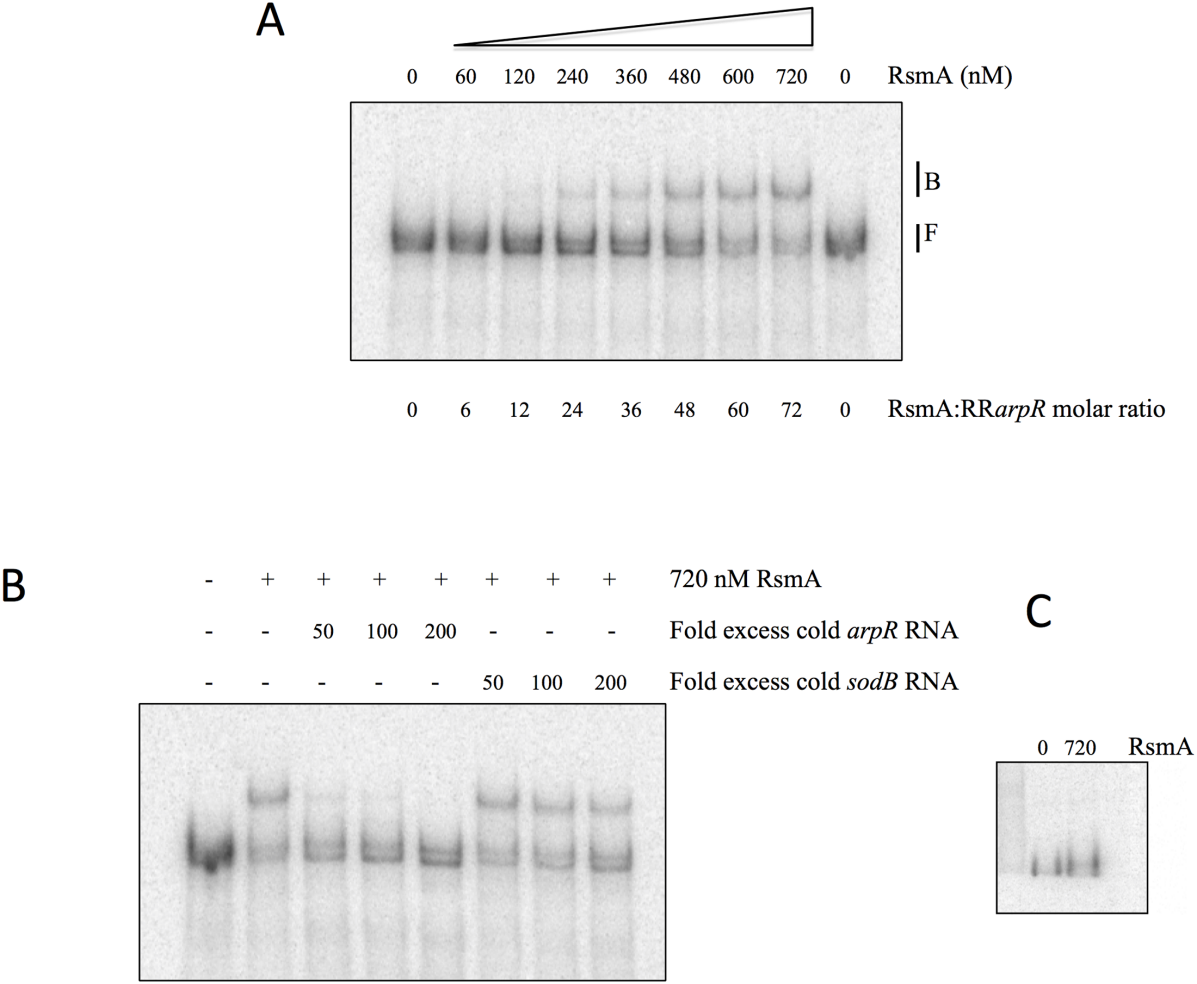
Binding of the RsmA protein to the mRNA of *arpR*. RNA gel mobility shift assay to analyze RsmA binding to the regulatory region of the *arpR* mRNA. (**A**) Labelled RNA fragments (10 nM) containing the regulatory region of *arpR* were incubated with increasing concentrations of His6-RsmA (0–720 nM). (**B**) The binding of RsmA to the *arpR* RNA was further analyzed by competitive EMSA. As a negative control, a fragment containing the regulatory region of the *sodA* RNA was included in the RNA binding reaction. The labelled RNA fragment containing the regulatory region of *arpR* was mixed with 720 nM of His6-RsmA in the presence or absence of up to 200-fold excess of unlabelled specific (*arpR*) or non-specific (*sodA*) competitor RNAs. **(C)** RNA gel mobility shift assay of an RNA fragment (10nM) containing the regulatory region of the *sodA* RNA (negative control) with the His6-RsmA protein (0 and 720 nM). The RNA-protein complexes are indicated (RsmZ1-RsmA, **B**; and RsmZ1 free, **F**) and were resolved in non-denaturing 6% polyacrylamide gels.

To further support the regulatory model proposed, we carried out an analysis of the *arpR* Shine Dalgarno (SD) region to identify potential RsmA binding sites that closely resembled the SELEX-derived binding site consensus sequence RUACAR**GGA**UGU (R is adenine or guanine) [24]. We identified three putative binding sites able to form hairpin structures containing a GGA sequence, two of them overlapping the SD sequence. To demonstrate binding of the RsmA protein to this region of the *arpR* mRNA, we generated by *in vitro* transcription a 290 nt RNA corresponding to the 5´ end of the *arpR* leader (RR*arpR*) and tested the binding of purified RsmA protein to this transcript in RNA gel mobility shift experiments. As shown in Fig 5A, the interaction of RsmA with the RR*arpR* transcript was observed when testing molar ratios of RsmA/RR*arpR* from 12 to 72. As specificity control we used unlabeled (cold) RR*arpR* RNA in competence experiments (Fig 5B). From 50 to 200-fold molar excess of unlabeled *RRarpR* prevented the electrophoretic shift mobility of the labelled RR*arpR*, showing a competition effect. A non-specific binding was ruled out by testing the effect of the nonspecific RNA transcript from the gene *sodB* (RR*sodB*) in the mobility shift assay [9]. When using 50 to 200-fold molar excess of unlabelled RR*sodB* / labelled RR*arpR*, no effect was observed in the electrophoretic mobility of the labelled RR*arpR*, indicating that the *sodB* RNA was not able to displace the bound RR*arpR* RNA and thus is unable to bind RsmA (Fig 5B).

### Inactivation of *rsmA* in the *gacA* mutant restored *arpR* expression but not AR production nor *arsA* expression

The results presented above suggest that in the *gacA* mutant, the lower amount of RsmZ1 RNAs produced allows the RsmA protein to bind the *arpR* mRNA, preventing its translation, which in turn diminishes transcription of the ARs biosynthetic operon *arsABCD*. Therefore, inactivation of *rsmA* in the *gacA* mutant should restore *arpR* expression and the production of ARs. To test this hypothesis we inactivated the *rsmA* gene in the *gacA* mutant and analyzed the level of transcripts of *arpR* and *arsA* in this *gacA-rsmA* double mutant. As expected, the absence of RsmA indeed restored and even increased the level of the *arpR* transcript (Fig 3), confirming the regulatory model proposed. Unexpectedly, neither the expression of *arsA* (Fig 2) nor ARs production (Fig 1) were reestablished in the double mutant.

### The GacA system regulates the level of ARs through and additional pathway that controls expression of *arsA* and is independent of ArpR, RsmA and the coinducer of ArpR

**Fig 6:**
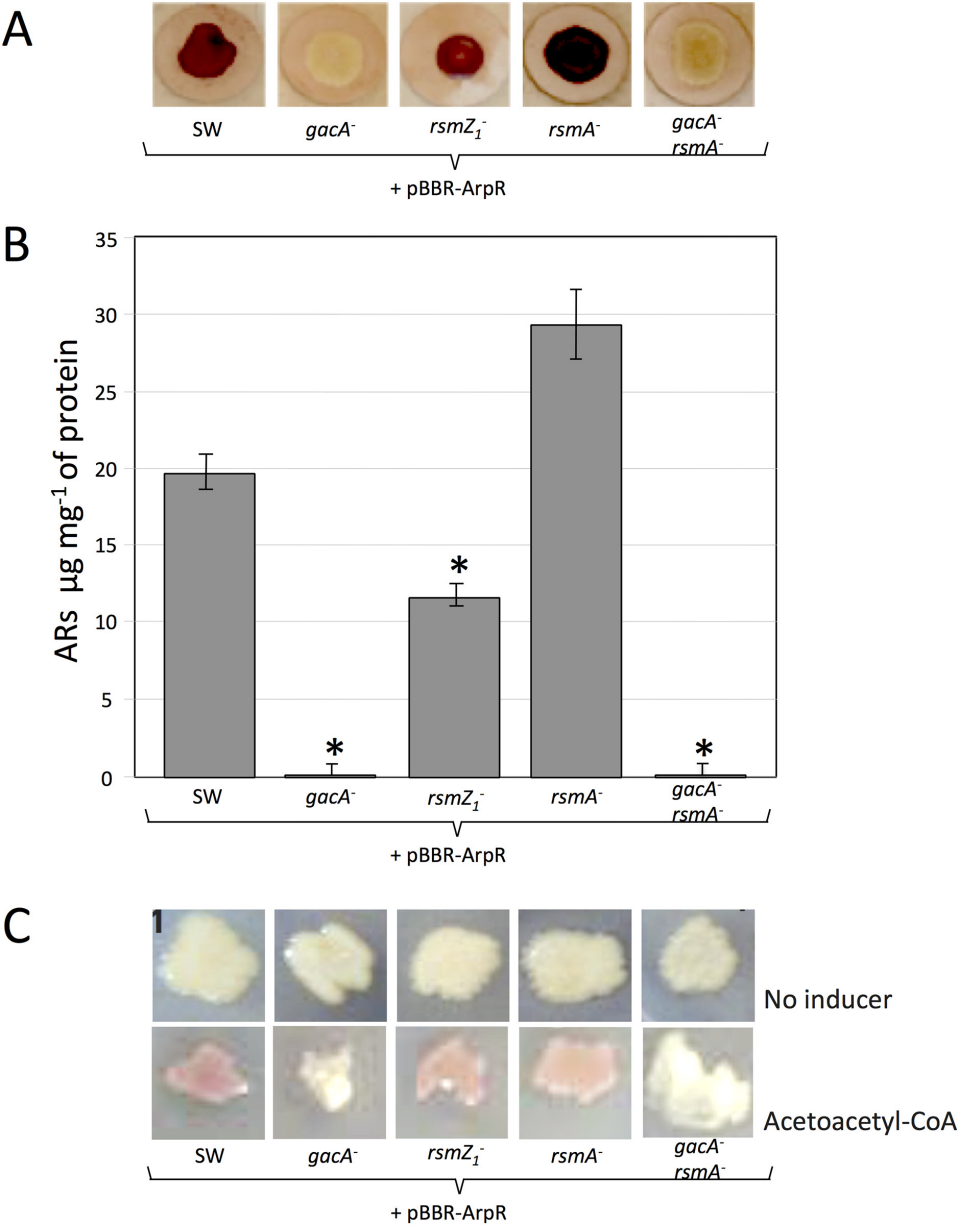
Effect of ArpR constitutive expression and acetoacetyl-CoA coinducer addition on ARs production of the mutant strains. **(A)** Staining with Fast Blue B of alkylresorcinols produced by colonies of *A*. *vinelandii* SW 136 wild type strain and its mutant derivatives *gacA*, *rsmZ1*, *rsmA and gacA-rsmA*. All strains were transformed with plasmid pBBR-ArpR, carrying a constitutively expressed *arpR* gene. The colonies were grown on Petri dishes under encystment inducing conditions for 5 days. **(B)** Quantification of the ARs produced by the same strains also expressing ArpR from plasmid pBBR-ArpR, under the same conditions shown in (A) Asterisks indicate statistical significance (unpaired Student's *t*-test) in the comparison of ARs content of each mutant versus the wild type strain, *P < 0.05. (C) Effect of the addition to the medium of acetoacetyl-CoA, the coinducer of ArpR, on ARs production visualized by staining with Fast Blue B. The colonies were grown on Petri dishes under vegetative conditions for 5 days in the absence or presence of 5 μM acetoacetyl-CoA. The stains were also transformed with plasmid pBBR-ArpR.

To further analyze if the control of GacA on ARs synthesis is exerted through the regulation of *arpR* expression, we expressed *in trans* the ArpR protein in the *gacA*, the *rsmZ1* and the double *gacA-rsmA* mutants. This ArpR protein, tagged with six Histidine residues at its N terminus, was expressed from a constitutive promoter using plasmid pBBR-ArpR [6] under encysting conditions. The expression of ArpR in all these strains was confirmed by Western blot analysis (not shown). As expected, expression of ArpR reestablished ARs production in the *rsmZ1* mutant and had no effect in the *rsmA* mutant (Fig 6A and 6B), confirming the regulation exerted by the Rsm system on ARs synthesis through the control of ArpR expression; however, the *gacA* and the double *gacA-rsmA* mutants expressing ArpR did not produce ARs, further supporting the hypothesis of an additional regulatory role of GacA on the expression of the ARs biosynthetic genes which is independent of the presence of ArpR.

Because the activation of *arsA* transcription by ArpR has been shown to depend on the presence of the coinducer acetoacetyl-CoA [6], we tested if the lack of ARs synthesis in the *gacA* and *gacA-rsmA* mutants expressing ArpR could be due to a deficient production of this molecule. For this experiment, all the strains were grown on BS with and without the coinducer and expressing ArpR from a constitutive promoter. Under this vegetative growth condition, neither strain was able to produce ARs in the absence of acetoacetyl-CoA (Fig 6C); however, in the presence of the coinducer, the wild type strain and the *rsmZ1* and the *rsmA* mutants produced ARs; however, the *gacA* and *gacA-rsmA* mutants were not stained. These phenotypes of ARs production were very similar to those obtained under encysting conditions. This indicates that the additional regulatory role of GacA on *arsA* expression and ARs production is probably not related with the production of the coinducer of ArpR.

These results indicate that GacA positively controls expression of alkylresorcinol biosynthetic genes through the regulation of the Rsm system and its negative posttrancriptional effect on *arpR* expression, but also by another pathway independent of the ArpR.

## Discussion

The Gac system regulates the biosynthesis of alginate and polyhydroxybutyrate, two polymers that are components of the *A*. *vinelandii* cyst. This control is exerted through the Rsm system, because GacA activates transcription of the small RNAs (RsmZs) that counteract the activity of the RsmA protein, a repressor of translation of the mRNAs of the alginate biosynthetic gene *algD* [10] and of *phbR*, that codes for the transcriptional activator of the PHB biosynthetic genes [9]. In *A*. *vinelandii* ATCC 9046 GacA also controls the expression of the alternative sigma factor RpoS [16], that in *A*. *vinelandii* SW136 is required for the expression of ArpR, the transcriptional activator of the ARs biosynthetic genes [6]. To find out if the synthesis of ARs is also controlled by the Gac or the Rsm regulatory systems, we tested the effect of inactivating the *gacA*, *rsmZ1* and *rsmA* genes on ARs production. The results indicate that GacA regulates ARs biosynthesis and that this regulation is through the control of Rsm, because the *gacA* and *rsmZ1* mutants produced less ARs (Fig 1) and had lower levels of *arsA* transcripts than the wild type strain, whereas the *rsmA* mutant overproduced ARs and had higher levels of *arsA* mRNA (Figs 1 and 2). Because the previous works that showed regulation of *rsmZ1* by GacA were done using vegetative cells [9, 10] we confirmed that GacA also regulates the expression of *rsmZ1* during encystment by showing that in *A*. *vinelandii* cysts, the inactivation of *gacA* impaired *rsmZ1* expression. The regulation by GacA through the control of *rpoS* expression in *A*. *vinelandii* SW136 was ruled out, because inactivation of GacA did not affect the level of transcripts of this sigma factor in this strain. This result was somewhat surprising, because it could be expected that the regulatory mechanisms controlling a process like encystment would be highly conserved in different strains of the same species. This difference in the regulation of *rpoS* is not understood, but it could be due to a divergence in the evolution of encystment in *A*. *vinenaldii* strains isolated from different places, and therefore subject to different selective pressures, or it could have been acquired during the manipulation of the bacterium in the laboratory, as was the case for *A*. *vinelandii* strain OP, a non-gummy mutant [25], which spontaneously acquired an insertion sequence interrupting the gene of the alternative sigma factor AlgU, making it unable to produce alginate [26].

Because the nitrogen-related phosphotransferase system and the alternative sigma factor RpoS control ARs synthesis by regulating the expression of ArpR [6, 8], we studied if this could also be the case for the regulation by GacA and the Rsm systems. We quantified the levels of *arpR* transcripts in the *gacA*, *rsmZ1* and *rsmA* mutants (Fig 3). The amounts of *arpR* mRNAs were affected by the mutations with a pattern similar to that observed for *arsA*, although to a minor extent, suggesting that the regulation was through *arpR*. Considering this result and given the effects observed on other genes regulated by the Rsm system, both on translation of the target mRNAs and on their stability, we reanalyzed the expression of *arsA* and *arpR* but using transcriptional and translational gene fusions (Fig 4). For *arsA*, the effects of the *gacA*, *rsmZ1* and *rsmA* mutations were almost identical when comparing the transcriptional and translational gene fusions. They also showed the same effects obtained by RT-qPCR (Fig 2). This result suggested that the effect on *arsA* is at the transcriptional level; however, for *arpR* the effects of the *gacA* and *rsmZ1* mutations were considerably stronger in the translational fusions. This result suggested a regulatory effect by RsmA on translation of *arpR*. The demonstration of the binding of the RsmA protein to the regulatory region of the *arpR* RNA (Fig 5) further supports the hypothesis of a negative translational effect by binding of this protein to the mRNA of *arpR*.

A confirmation of the role of GacA and RsmA on *arpR* regulation is provided by the analysis of the effect of inactivating *rsmA* in the *gacA* mutant. In this double mutant the expression of *arpR* is completely reestablished (Fig 3), according to the model proposed. Also, the reestablishment of ARs production in the *rsmZ1* mutant by expressing ArpR from a constitutive promoter (Fig 6) further supports our model; however, in the double mutant *gacA-rsmA* neither ARs production nor the expression of *arsA* were reestablished, and the constitutive expression of ArpR in the *gacA* or the *gacA-rsmA* doble mutants did not reestablish ARs synthesis, showing an additional regulation by GacA on *arsA* expression independent of ArpR and the Rsm system. This regulation is not related with the presence of the ArpR coinducer, because the addition of acetoacetyl-CoA did not allow ARs production in the strains containing the *gacA* inactivation.

**Fig 7:**
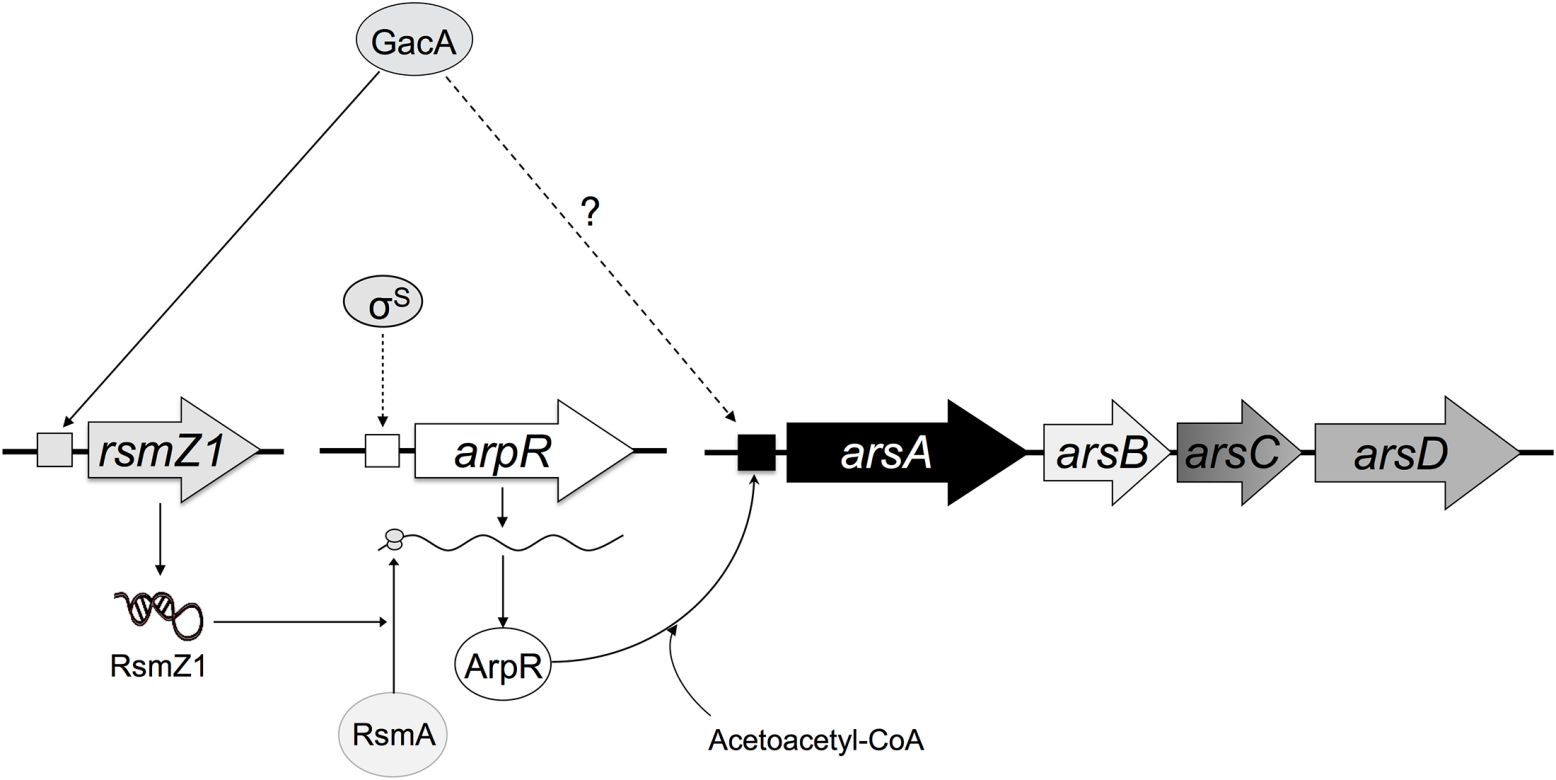
Proposed model for the control of ARs synthesis by the Gac-Rsm system in *A*. *vinelandii* SW136. The regulatory protein GacA controls ARs synthesis by two different pathways, one dependent on the Rsm system, that regulates the expression of the transcriptional activator ArpR, and a second pathway that positively controls expression of the *ars* biosynthetic genes independently of these regulators. Dashed lines indicate unknown intermediates or unknown mechanism of regulation.

The results presented are compatible with a regulatory model (Fig 7) where the RsmA protein would be a negative translational regulator of *arpR* by binding its mRNA, thus affecting transcription of the *arsABCD* biosynthetic genes. The RsmZ1 small RNA would be titrating the RsmA protein, blocking its negative effect. Inactivation of *rsmZ1* would leave RsmA free to exert its negative effect mainly on translation of the *arpR* mRNA. Because GacA activates transcription of *rsmZ1*, its absence would cause a similar effect to that of the *rsmZ1* inactivation. Also, in the absence of GacA or RsmZ1, the stability of the *arpR* transcripts would be negatively affected, as has been demonstrated for other other target mRNAs in *A*. *vinelandii* [9]. According to our results, GacA would have an additional positive regulatory role on *arsABCD* expression, independent of ArpR and RsmA.

Under vegetative growth conditions, transcription of the *rsmZ1-7* and *rsmY* genes, whose RNAs interact with RsmA, was previously shown to be induced to different levels in the stationary phase and to be dependent on GacA [9]. The effects of the *rsmZ1* mutation on expression of *arpR* and ARs synthesis suggest that, under encystment inducing conditions, the expression of RsmZ1 or its activity must be favored over the other RNAs. ß-glucuronidase activity in strains carrying transcriptional *gusA* gene fusions of *rsmZ1*, *rsmZ2*, *rsmZ3*, *rsmZ4*, *rsmZ5*, *rsmZ6*, *rsmZ7* and *rsmY* showed that under encysting conditions all these RNAs are expressed (data not shown). This result suggests that RsmZ1 is the main Rsm RNA active against RsmA under encysting conditions by an unknown regulatory mechanism that remains to be investigated.

## Supporting Information

S1 Fig Effect of inactivation of *gacA* on the expression of *rsmZ1* under vegetative growth (A), and under encystment conditions (B). The expression levels of *rsmZ1* at different times were quantified as ß-glucuronidase activity of strains containing an *rsmZ1*::*gusA* transcriptional fusion. **Black bars** represent the expression levels of a SW136 wild type derivative strain containing an *rsmZ1*::*gusA* gene fusion (strain YRR62). The **grey bars** show *rsmZ1* expression in a *gacA* mutant derivative with the same *rsmZ1*::*gusA* gene fusion (strain YRR63). For vegetative growth (A) the cells were grown on Burk sucrose medium and for encystment induction (B) the cells were incubated in Burk´s medium with 0.2% butanol. One unit of ß-glucuronidase corresponds to 1 nmol of substrate (X-Gluc) hydrolyzed min^-1^ mg Protein^-1^. Determinations in both panels were made from bacterial cultures grown in flasks at 30°C.
(TIFF)

S1 Table Plasmids and strains used in this study. (DOCX)
