# Supplementary figures and images for: The GacS/A-RsmA Signal Transduction Pathway Controls the Synthesis of Alkylresorcinol Lipids that Replace Membrane Phospholipids during Encystment of Azotobacter vinelandii SW136
Source: PLoS One. 2016 Apr 7;11(4):e0153266. doi: 10.1371/journal.pone.0153266 (PMC4824345; doi:10.1371/journal.pone.0153266)

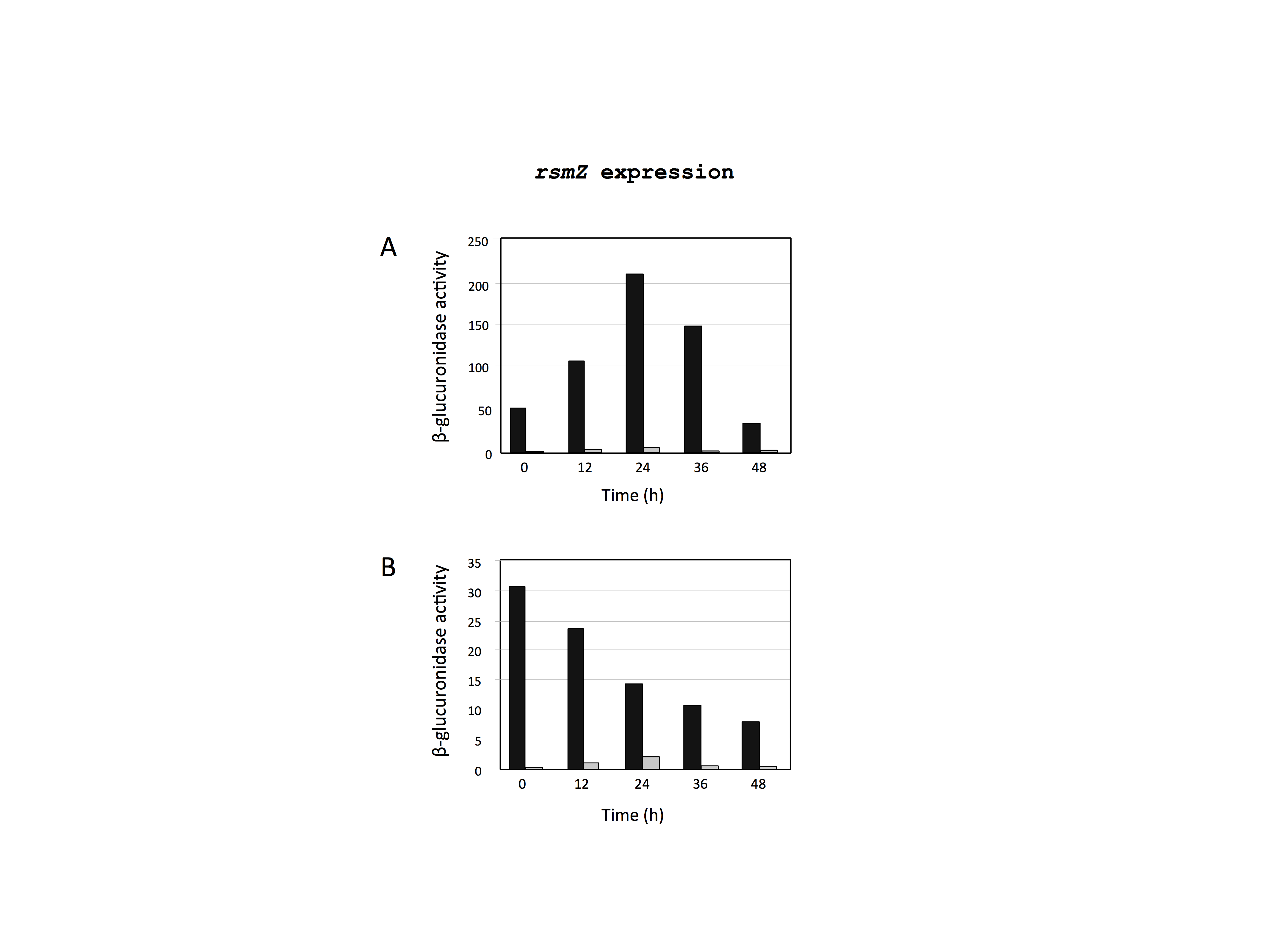

Supplement: S1 Fig — The expression levels of rsmZ1 at different times were quantified as ß-glucuronidase activity of strains containing an rsmZ1::gusA transcriptional fusion. Black bars represent the expression levels of a SW136 wild type derivative strain containing an rsmZ1::gusA gene fusion (strain YRR62). The grey bars show rsmZ1 expression in a gacA mutant derivative with the same rsmZ1::gusA gene fusion (strain YRR63). For vegetative growth (A) the cells were grown on Burk sucrose medium and for encystment induction (B) the cells were incubated in Burk´s medium with 0.2% butanol. One unit of ß-glucuronidase corresponds to 1 nmol of substrate (X-Gluc) hydrolyzed min-1 mg Protein-1. Determinations in both panels were made from bacterial cultures grown in flasks at 30°C. (TIFF) [file pone.0153266.s001.tiff]
