# Supplementary material for: The GacS/A-RsmA Signal Transduction Pathway Controls the Synthesis of Alkylresorcinol Lipids that Replace Membrane Phospholipids during Encystment of Azotobacter vinelandii SW136
Source: PLoS One. 2016 Apr 7;11(4):e0153266. doi: 10.1371/journal.pone.0153266 (PMC4824345; doi:10.1371/journal.pone.0153266)
Supplement: S1 Table — (DOCX) [file pone.0153266.s002.docx]

**S1 Table.** Plasmids and strains used in this study.

| Strain or Plasmid | Description | Reference or source |
| --- | --- | --- |
|  |  |  |
| *Plasmids* |  | |
|  |  |  |
| pARRA | pTZ19R with *arpR* regulatory region under the T7 promoter. | This study |
| pBBR-ArpR | pBBR1MCS-2 derivative expressing *ArpR-His* gene under the km promoter. | [6] |
| pLM1 | pTZ19R with the *sodB* regulatory region under the T7 promoter. | [9] |
| pMOS*Blue* | Plasmid for cloning PCR products. | Amersham |
| pMOSgA | pMOS*Blue* derivative carrying *gusA::Sp* cassette with no RBS. | This study |
| pMP01 | pBSL97 derivative with a Sp-*gusA* cassette. | [17] |
| pYG1 | pBADTOPO for expression of the *rsmA* gene. | [10] |
| pYRR40 | pMOS*Blue* derivative carrying 2.6 kb fragment containing arsA. | [6] |
| pYRR41 | pMOS*Blue* derivative carrying 1.8 kb fragment containing arpR. | [6] |
| pYRR50 | pMOS*Blue* derivative carrying an *arsA::gusA* transcriptional fusion. | [6] |
| pYRR51 | pMOSBlue derivative carrying an arsA::gusA transcriptional fusion. | This study |
| pYRR52 | pMOS*Blue* derivative carrying an *arpR::gusA* translational fusion. | [6] |
| pYRR53 | pMOSBlue derivative carrying an arpR::gusA translational fusion. | This study |
| pTZ19R | Cloning vector with T7 promoter for *in vitro* transcription assays. | ThermoScientific |
| *A. vinelandii srains ATCC 9046* | Wild type, highly mucoid. | ATCC |
| JM3 | ATCC 9046 derivative with a *gacA*::Gmr mutation. | [16] |
| AHrsmA | Mutant derivative of UW136 with an *rsmA*::Spr insertion. | [9] |
| AEIV*rsmZ1* | Mutant derivative of strain AEIV with an rsmZ1::Kmr insertion. | [10] |
| SW136 | *algU*+ mucoid derivative of UW136. | [2] |
| SW5 | SW136 with a *gacA*::Gmr insertion. | This study |
| SW11 | SW136 with an *rsmA*::Spr insertion. | This study |
| SW13 | SW136 with an *rsmZ1*::Kmr insertion. | This study |
| SW15 | SW5 with an *rsmA*::Spr insertion. | This study |
| YRR30 | SW136 with a chromosomal *arsA–gusA*-Spr transcriptional fusion. | [6] |
| YRR31 | SW136 with a chromosomal *arsA–gusA*-Spr translational fusion. | This study |
| YRR33 | SW 5 with a chromosomal *arsA–gusA* -Spr translational fusion. | This study |
| YRR36 | SW 5 with a chromosomal *arsA–gusA* -Spr transcriptional fusio.n | This study |
| YRR38 | SW11 with a chromosomal *arsA–gusA*-Spr transcriptional fusion. | This study |
| YRR39 | SW11 with a chromosomal *arsA–gusA*-Spr translational fusion. | This study |
| YRR40 | SW13 with a chromosomal *arsA–gusA*-Spr transcriptional fusion. | This study |
| YRR41 | SW13 with a chromosomal *arsA–gusA*-Spr translational fusion. | This study |
| YRR50 | SW136 with a chromosomal *arpR–gusA*-Spr translational fusion | [6] |
| YRR51 | SW136 with a chromosomal *arpR–gusA*-Spr translational fusion. | This study |
| YRR53 | SW 5 with a chromosomal *arpR–gusA* -Spr translational fusion. | This study |
| YRR54 | SW 5 with a chromosomal *arpR–gusA* -Spr transcriptional fusion. | This study |
| YRR56 | SW11 with a chromosomal *arpR–gusA*-Spr transcriptional fusion. | This study |
| YRR57 | SW11 with a chromosomal *arpR–gusA*-Spr translational fusion. | This study |
| YRR58 | SW13 with a chromosomal *arpR–gusA*-Spr transcriptional fusion. | This study |
| YRR59 | SW13 with a chromosomal *arpR–gusA*-Spr translational fusion. | This study |
| YRR62 | SW136 with a chromosomal *rsmZ1*–*gusA*-Spr translational fusion. | This study |
| YRR63 | SW5 with a chromosomal *rsmZ1*–*gusA*-Spr transcriptional fusion. | This study |
